# Enhancing Teacher Gatekeeper Skills for Suicide: A Cluster-Randomized Controlled Trial Among School-Based Lay People in China

**DOI:** 10.21203/rs.3.rs-4540562/v1

**Published:** 2024-11-15

**Authors:** Runsen Chen, Diyang Qu, Bowen Liu, Xuan Zhang, Chengxi Cai, Dongyang Chen, Dongyu Liu, Xue Wen, Zhijun Wu, Jing An, Shufang Sun, Shekhar Saxena

**Affiliations:** Tsinghua University; Tsinghua University; Tsinghua University; Tsinghua University; Tsinghua University; Tsinghua University; School of Public Health, LKS Faculty of Medicine, The University of Hong Kong; Tsinghua University; Tsinghua University; Tsinghua University; Brown University School of Public Health; Harvard

## Abstract

Gatekeeper training equips individuals with the skills to identify those exhibiting warning signs of suicide risk and refer them to appropriate services. However, enhancing gatekeepers’ knowledge, efficacy, subsequent behaviors, and the broader implications of such interventions in school settings remain pressing concerns. To address these challenges, the Life Gatekeeper Training Program (LGTP) was developed. This 8-session program is designed to train school teachers in essential gatekeeper skills through case demonstrations, role-plays, and group discussions, by using a train-the-trainer model. A cluster randomized controlled trial, which randomly assigned 84 schools (including 223 trainers and 4,140 trainees) to intervention and waitlist control groups, was conducted from December 2022 to March 2024 to evaluate the 6- and 12-month outcomes of the LGTP in Yunfu, China. The primary outcomes were teachers’ stigmatization, suicide literacy, perceived competence, and willingness to intervene. The secondary outcome measures were gatekeeper behaviors, including identifying students at risk, discussing potential suicide risk with them, or with their caregiver(s), and referring them to a mental health professional or a specialist clinic. The study was pre-registered with the Chinese Clinical Trial Registry, with a registration number of ChiCTR2200066142. Following the principle of intention to treat (ITT), the results of the generalized estimating equations showed LGTP intervention led to significant decreases in stigmatization *(b* = − 0.80, SE=0.04, *P*_FDR_ 0.001), increases in suicide literacy *(b* = 0.98, SE=0.04, *P*_FDR_<0.001), perceived competence *(b* = 1.03, SE=0.04, *P*_FDR_<0.001), and willingness to intervene *(b* = 0.76, SE=0.04, *P*_FDR_<0.001) compared to the control group at post-intervention, and these effects were moderately maintained at the 6 and 12 months follow up. In addition, gatekeeper behavior outcomes (i.e., Identify risk students, Talk to the students, Talk to the parents, Refer to professional help) in the intervention group were significantly higher than those in the control group. The LGTP, a standardized program with a brief training format, demonstrated efficacy in increasing actual gatekeeper behaviors among school teachers in China. The delivery strategies of this program enabled rapid scalability to reach a large population within a short time frame, thereby offering opportunities to expand early intervention and prevention efforts.

## Introduction

Gatekeeper training programs^1^, are designed to equip individuals with the skills to recognize warning signs of suicide, practice specific communication techniques^2^, and facilitate the timely identification and referral of at-risk individuals to mental health professionals^3^. Interestingly, the purpose of the gatekeeper program is similar to that of Cardiopulmonary Resuscitation (CPR) training, as both aim to reach as many community members as possible to equip them with basic skills to help during the critical period^4^, without turning them into crisis management experts.

However, the school-based gatekeeper program remains underexplored, as a recent review highlighted that gatekeeper programs at school effectively increased knowledge^5^—the primary outcomes of majority gatekeeper programs—the effectiveness of such training in improving skills, the likelihood of intervention, and actual gatekeeper behaviors remains unclear^6^. In addition, the findings indicated that the majority of published studies have focused on high-income countries, such as the US, Portugal, and Germany (93%). These studies typically had relatively small sample sizes (N = 204), short observation periods (average = 3 months), and presented mixed findings, often lacking assessments of behavioral changes. Specifically, 73% of the studies employ single-arm studies or control groups without pre-tests, and none showed significant improvement in gatekeeper behaviors^5^. This current situation raises several questions, including to what extent school-based programs can improve gatekeepers’ actual behaviors, addressing the ‘know-to-do gaps’^7,8^which is a critical step before impacting real suicide rates. In addition, how these programs can be adopted and implemented in different cultural settings also remains a significant question. According to the Cultural Theory of Suicide, culture influences the types of stressors that contribute to suicide and the ways suicidal ideation, plan and behaviors are expressed. Therefore, previous gatekeeper programs designed for other cultures, particularly those emphasizing specific risk factors and warning signs, may not be directly applicable to different cultural contexts^9^.

China, a home of over 293 million students^10^, highlighted the importance of school-based mental health intervention programs^11^. In addition, governments at national level have been actively involved in promoting these initiatives, providing policy support to ensure that schools can address the mental health needs of students effectively. For example, the Ministry of Education of the People’s Republic of China has issued a Notice emphasizing that when students experience psychological crises, including suicidal tendencies, schools must promptly assist parents in ensuring the students are taken to a hospital for further help^12^. Local governments also provide detailed procedures. For example, the Department of Education of Guangdong Province has published a ‘School Safety Regulations of Guangdong Province’ and highlighted that, if schools find the students has an obvious suicide risk, they must take necessary measures such as monitoring, accompanying, and inform their guardians or other close relatives in a timely manner^13^. Therefore, training school staff to enhance their ability to identify and refer at-risk individuals is highly necessary.

However, questions remain regarding how to localize such programs, who should be trained, and how the training should be effectively implemented^14^. In China, in addition to mental health teachers, other roles have also been proposed to enhance the effectiveness of these interventions. Class room teachers or head teachers *(“Ban Zhuren”),* in alignment with the Head Teacher System in China, may be a key. In this system, head teachers are mandated in every elementary and middle school class and are responsible for gaining a comprehensive understanding of each student, managing the class’s daily activities, overseeing student mental health, and frequently communicating with other faculty and staff. This supervisor role could potentially address the shortage of professional mental health staff in such areas and may even enhance the efficacy of the training, as head teachers interact with the students throughout the day. This strategy is also in line with practices in other countries, such as Pakistan and India, where general class teachers are also trained to recognize warning signs of emotional disorders in students^15,16^.

Taken together, we developed a localized, 8-session school-based gatekeeper training program, entitled the Life Gatekeeper Training Program (LGTP)^17^. The whole program consisted of five stages. In stage 1, which involved developing training materials, over 31 nationwide experts were enlisted to participate in a two-round Delphi process, resulting in the inclusion of 201 statements regarding intervention components^17^. For example, the session included how to communicate with parents were added after the Delphi stage. Stages 2 and 3 were pilot stages for implementing the program in local schools. The initial pilot study utilized a face-to-face training approach but encountered difficulties in scaling up quickly in real-world settings. Consequently, the training format was revised to a digital-based version, with all necessary teaching materials and activities pre-recorded.

In this stage, in order to test the efficacy of this finalized program, we conducted a large-scale randomized controlled trial (RCT) in Yunfu, China, and the intervention package was delivered to 42 schools using a Train-the-Trainer approach^18^. Specifically, the LGTP was initially delivered online by specialized professionals to 2 to 3 trainer teachers (i.e., mental health teachers) recruited from each school. These trainer teachers then conducted eight sessions of the program face-to-face with future trainees (i.e., head teachers), following standardized guidelines. The outline and guideline were provide in supplementary materials. The primary hypothesis was that the LGTP would significantly reduce suicide-related stigma among teachers, enhance their literacy regarding suicide, and further improve their willingness and competence to intervene with students at risk.

## Results

**Figure 2.**
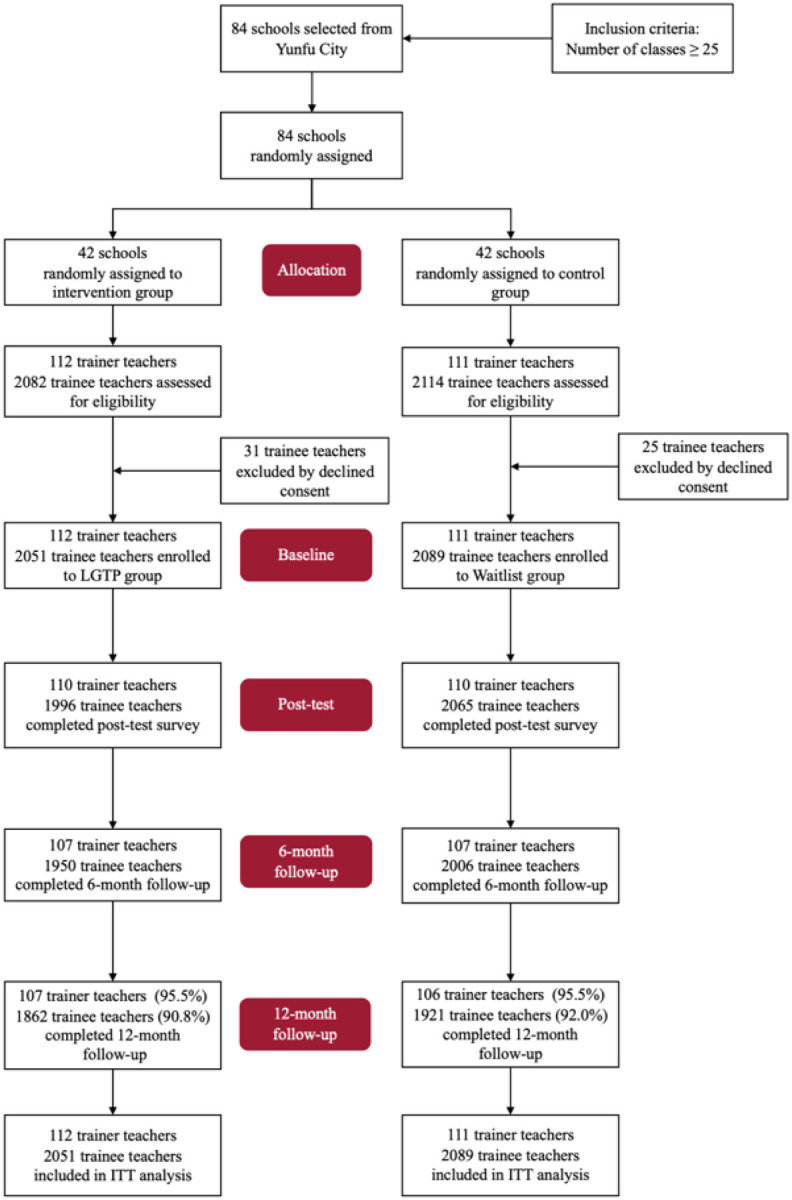
Trial profile **Notes**: LGTP = Life Gatekeeper Training Program; Waitlist control; ITT=intention-to-treat.

In December 2022, a total of 4363 participants were recruited into the trial, including 112 trainer teachers and 2051 trainee teachers from 42 intervention schools, and 111 trainer teachers and 2089 trainee teachers from 42 waitlist-control schools included in the ITT analysis (Figure 2). The last 12-month follow-up visit was conducted in March 2024. During the 12 months, 194 participants in the intervention group and 173 participants in the control group were lost to follow-up, resulting in a retention rate of 91.6%. The overall proportion of missing data is below 5%.

Table 1 summarizes the demographics and baseline characteristics of participants. On average, within the intervention group, trainer teachers were 36.4 years old (SD 8.6), with 72 (64.3%) female, and trainee teachers were 37.2 years old (SD 8.8), with 1536 (74.9%) female. Meanwhile, in the control group, trainer teachers averaged 36.0 years old (SD 8.5), with 73 (65.8%) female, and trainee teachers averaged 36.9 years old (SD 8.7), with 1556 (74.5%) female. No significant differences were found between conditions in demographic variables (age, gender, etc.) at baseline for both trainers and trainees (*p* > 0.05). No adverse events or harms were observed in either group during the study period (See Supplementary Materials for more details regarding the process).

### Primary outcomes

Trainer teachers (i.e., the psychological health education teachers who were first trained in step 1 of the TTT format) showed significantly better outcomes in intervention group than in control group (see details in supplementary table 1–2). For primary outcomes of trainee teachers (i.e., the lay head teachers who were then trained in step 2 of the TTT format), the GEE model and t-test results are presented in table 2 and 3. Table 2 shows the interactions between the groups and time on the four primary outcomes using the GEE model after controlling for covariables such as age, gender, education level, family income, religion, years of work, and baseline outcomes scores. The results indicate that LGTP intervention significantly reduced stigma against suicide at T2 (*b* = − 0.80, SE=0.04, *P*_FDR_ 0.001), T3 (b= − 0.45, SE=0.04, *P*_FDR_ 0.001), and T4 (b= − 0.32, SE=0.04, *P*_FDR_ 0.001) compared to the control group. Additionally, the intervention significantly increased suicide literacy at T2 (*b* = 0.98, SE=0.04, *P*_FDR_ 0.001), T3 (*b* = 0.57, SE=0.04, *P*_FDR_ 0.001), and T4 (*b* = 0.50, SE=0.04, *P*_FDR_ 0.001) relative to the control group. Furthermore, the intervention significantly increased the perceived competence at T2 (*b* = 1.03, SE=0.04, *P*_FDR_ 0.001), T3 (*b* = 0.52, SE=0.04, *P*_FDR_ 0.001), and T4 (*b* = 0.46, SE=0.04, *P*_FDR_ 0.001) compared to the control group. Lastly, the intervention significantly increased the willingness to intervene at T2 (*b* = 0.76, SE=0.04, *P*_FDR_ 0.001), T3 (*b* = 0.40, SE=0.04, *P*_FDR_ 0.001), and T4 (b= 0.40, SE=0.04, *P*_FDR_ 0.001) in comparison to the control group. The details about the simple effects of *time* and *group* fortrainee teachers are presented in the supplementary table 3–6.

**Figure 3.**
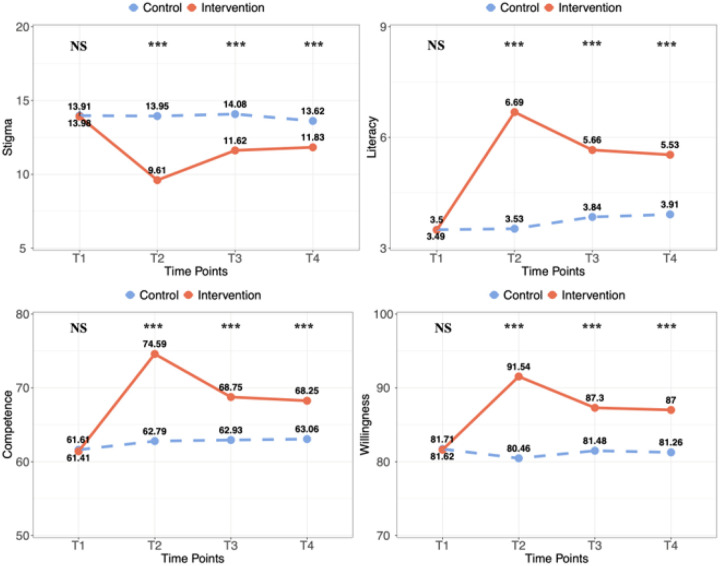
Indicated the change in primary outcomes between two groups over 12 months among trainee teachers. Note: T1: Baseline; T2: Post-test; T3: 6-month follow-up; T4: 12-month follow-up The results of trainee teachers are presented in the supplementary table 6.

Table 3 and Figure 3 further show the changes in the means of the four primary outcomes. Compared to the waitlist control, the intervention group had significant improvement on stigma against suicide immediately post-intervention (Cohen’s *d* = −0.85, *P*_FDR_ 0.001), and this effect was maintained at 6 months (Cohen’s *d*= −0.49, *P*_FDR_ 0.001) and 12 months post-intervention (Cohen’s *d*= −0.34, *P*_FDR_ 0.001). Similarly, the intervention group had significantly improvement on suicide literacy compared to control condition at immediately post-intervention (Cohen’s *d*= 1.05, *P*_FDR_ 0.001) and this effect was maintained at 6 months (Cohen’s *d* = 0.59, *P*_FDR_ 0.001) and at 12 months (Cohen’s *d* = 0.52, *P*_FDR_ 0.001). Additionally, perceived competence was significantly improved in the intervention group compared to those in the control group at immediately post-intervention (Cohen’s *d* = 1.00, *P*_FDR_ 0.001), 6 months follow-up (Cohen’s *d* = 0.53, *P*_FDR_ 0.001), and 12 months follow up (Cohen’s *d* = 0.47, *P*_FDR_ 0.001). Similarly, compared to the waitlist control, participants in the intervention condition had significantly improved willingness to intervene at immediately post-intervention (Cohen’s *d* = 0.78, *P*_FDR_ 0.001), 6 months follow-up (Cohen’s *d* = 0.40, *P*_FDR_ 0.001), and 12 months follow-up (Cohen’s *d* = 0.39, *P*_FDR_ 0.001).

### Secondary Outcomes

As shown in **Table 4**, the gatekeeper behavior outcomes, measured at 12 months follow-up, were significantly different between the groups. Specially, intervention group participants identified a total of 2,096 students at suicidal risk during the 12-month follow-up, while 1,335 students were identified in the control group. In the intervention group, 37% of the identified students were talked to (significantly higher than the control group, which was 22%, *t*= 10.80, *P*_FDR_ 0.001), 37% were further intervened by talking with their caregivers (significantly higher than the control group, which was 22%, *t* = 11.26, *P*_FDR_ 0.001), and 20% were referred to professional help (significantly higher than the control group, which was 12%, *t* = 7.31, *P*_FDR_ 0.001). The gatekeeper behavior outcomes of trainer teachers and the 6-month follow-up results of the intervention group’s trainee teachers are provided in supplementary table 7–8.

### Satisfaction

Overall satisfaction with the program was measured through post-intervention surveys with teachers. In total, teachers reported high levels of satisfaction across multiple level: they rated their overall satisfaction with the training highly (Trainer teachers: M = 4.78, SD = 0.63; Trainee teachers: M = 4.52, SD = 0.76), demonstrated a strong mastery of the content (E2) (Trainer teachers: M = 4.26, SD = 0.66; Trainee teachers: M = 3.97, SD = 0.77), indicated that they gained knowledge from the training (Trainer teachers: M = 4.66, SD = 0.55; Trainee teachers: M = 4.19, SD = 0.79), and expressed a strong willingness to recommend the training to other teachers (Trainer teachers: M = 4.86, SD = 0.39; Trainee teachers: M = 4.36, SD = 0.77).

## Discussion

The LGTP, an 8-session intervention, demonstrated significant improvements compared to the waitlist control group in reducing gatekeepers’ self-reported stigma toward suicide, enhancing participants’ knowledge of suicide, increasing their willingness and competence to intervene, and fostering gatekeeper behaviors in identifying and responding to at-risk children and adolescents. These changes remained significant and of similar magnitude at one year, highlighting the long-lasting effects of this brief training. These findings underscore its potential for broader implementation and scalability within general school systems, particularly in real-world contexts where gatekeeper training for teachers is urgently needed to address suicide prevention among students.

The increased knowledge and reduced stigma against suicide among teachers may enable them to overcome barriers in identifying at-risk students^19^, which is crucial premise for gatekeeper behaviors. These findings align with studies in a recent systematic review in overcoming the barrier caused by at-risk students’ hesitation to seek help, as previous studies found less than one-third of those with suicidal thoughts reached out for support^20,21^. Going beyond knowledge and attitude changes identified in prior research, the LGTP also demonstrated that non-specialist school teachers significantly improved their sense of competence and willingness to intervene both in the short and long term, translating knowledge into more readily and needed actions. This underscores the significant potential of this standardized, video-based training for scalability, and may address concerns about its effectiveness compared to in-person training.

The significant increase in participants’ gatekeeper behaviors during the follow-up periods in this study paves its way for gatekeeper training as a suicide prevention strategy. To date, only a few published studies on school-based gatekeeper programs have presented findings regarding behavioral changes^6^. For example, one study reported increased gatekeeper behaviors at three months follow-up among nearly five hundred students, college staffs, and faculty who attended a 90-minute training^22^. However, another short-term follow-up study revealed that changes in knowledge and efficacy did not translate into behavioral changes among college resident advisors as gatekeepers^23^. Similarly, two large-sample studies found no significant difference between intervention and control groups in promoting gatekeeper behaviors at both short- and long-term follow-ups after training teachers and other school personnel^24^.

One possible explanation for the effectiveness of the LGTP is its balanced approach to program duration and skill training, tailored to broaden the behavioral repertoire of participants. This may serve as a key mechanism for behavioral transformation, based on the highly recommended gatekeeper training approach exemplified by other brief training programs^22^. For instance, the program utilized role-play as a dynamic learning method, where participants started by observing role plays demonstrated by trainers. Subsequently, participants formed triads and took turns to practise being a gatekeeper, a student at risk of suicide, or an observer, allowing them to apply the skills in practice. This approach enabled participants to rehearse their skills across multiple hypothetical scenarios and benefit from immediate, personalized feedback during interactive sessions with fellow trainees. These dynamic interactions and behavior-focused practices have shown to be beneficial in enhancing skill acquisition and application, ultimately making participants more competent and confident in undertaking suicide prevention efforts^25^. Further, the used of teachers as trainers in LGTP may also have enhanced its community buy-in, ease of learning, transfer of knowledge, and perceived applicability, compared to traditional training approach via mental health professionals.

In addition, the decline in intervention effects observed at the 12-month follow-up warrants attention, even though the outcomes remain significantly improved compared to baseline. This finding aligns with previous studies, which also reported a reduction in outcomes over time^26,27^. It suggests that the initial benefits from gatekeeper training may diminish without continued reinforcement. Due to the limited opportunities for newly trained gatekeepers to apply their acquired knowledge and skills, retention may decline over time. Therefore, booster session after six months is recommended for effective gatekeeper training, such as through video applications or web-based interactive practice opportunities, as this method have demonstrated success in maintaining treatment-induced behavior changes^28^.

Our study has some limitations. First, the measures used to evaluate the program’s effectiveness are self-reported questionnaires, which may be influenced by social desirability^29^. Second, the generalizability of the results required further investigation, as the findings from the RCT may not be fully applicable in different cultural settings, especially since suicide exhibits cultural variations^9^. In addition, this training might create the misconception that mental health responsibilities are being shifted onto general education teachers, adding to their already demanding workload, rather than providing an additional opportunity of learning. Thus, further education of the aims for gatekeeper program were required. Last but not least, as a teacher-focused RCT, we did not specifically collect data on suicide risk observed among students from the 84 schools as outcomes due to the project’s aim to improve gatekeeping efficacy and behavior. However, future research should consider monitoring these events in the short and long term to assess the program’s impact on reducing suicide rates among adolescents in the real world. In addition, there is a potential for a placebo effect in the waiting list control group in this study design. This effect could potentially impact the validity of the comparison between the intervention and control groups. To address this, future studies might consider alternative control designs, such as active control groups or a crossover design, to mitigate the impact of the placebo effect.

## Conclusion

In conclusion, this large scale RCT provides empirical evidence of LGTP, a brief, universal school-based training targeting non-specialist school teachers, demonstrated its effectiveness of as a strategic approach to adolescent suicide prevention in rural China. By enhancing teachers’ knowledge, competency, and willingness to engage in gatekeeping, such programs could significantly contribute to improving their gatekeeper behaviors that may have crucial impact in addressing and preventing adolescent suicide. Our findings call for future actions for broader adoption and integration of gatekeeper training programs into school systems in resource-limited, rural settings.

## Methods

### Study design

A two-arm, non-blinded cluster RCT with two parallel groups (wait-list control and LGTP intervention), was conducted in schools in Yunfu City, Guangdong Province, China. Yunfu has over 2.3 million residents, 51.73% were male, and 19% of the population has an education level of high school or above, according to the government report published in 2021^30^. According to the 2023 report, Yunfu ranked last in terms of GDP among all cities in Guangdong Province, with a total GDP of less than 1207.42 billion RMB. This is significantly lower compared to Shenzhen (33398.98 billion RMB), the top city in the province^31^, and per capita GDP^32^ is approximately 170% lower than average number^33^. The details of study design were covered in our previously published articles, including a Delphi study^17^, pilot studies^34^, and a study protocol^35^. The current study was pre-registered with the Chinese Clinical Trial Registry, with a registration number of ChiCTR2200066142. The CONSORT-SPI 2018 checklist were provided in supplementary materials.

### Participants and intervention procedures

Participants, including all trainer teachers and trainee teachers, were recruited and enrolled in December 2022, and followed-up by March 2024. To ensure research efficiency, all schools with 25 or more classes in Yunfu were enrolled to achieve the efficacy of concentrating resources and obtaining a statistically significant sample size, which enhances the reliability and validity of the research findings. A total of 84 eligible schools were included in the study, including full-time teachers who participated in the study. Those who had previously participated in any systematic gatekeeper program training or declined to consent were excluded. All eligible teachers from the same school were randomly assigned to either the intervention group or the control group using cluster sampling. All participants were asked to provide informed consent before enrollment and were told that they could withdraw at any time. The data gathered was kept confidential.

In the intervention group, teachers were trained using a method referred to as the TTT model. In the LGTP, professional instructors first conduct primary online training for the intervention group’s trainer teachers who are the mental health teachers at schools. During the week following the primary training, trainer teachers from the intervention group conducted secondary face-to-face training for trainee teachers who are head teachers and do not specialize in mental health. To ensure adherence to the intervention protocol, instructors and trainer teachers conducted fidelity checks during each training session (see the protocol in supplementary materials)^36^. In the waitlist control group, schools continued their usual activities and training without any restrictions.

Each trainer teacher and trainee teacher completed the baseline survey (T1) one day before either the primary or secondary training of the intervention group, and the post-test survey (T2) immediately after the respective training. Follow-up surveys were administered 6 months (T3) and 12 months (T4) after T2. Surveys at all four time points were conducted simultaneously for both the control and intervention groups. Data collection training was provided to research team members before the study, and trainer teachers in two groups were also trained to assist the research team in data collection at their respective schools. There were no additional benefits or incentives provided to participating teachers, as stated in the informed consent form. However, the trained teachers received the certification after finished this program.

### Sample Size Calculation

Sample size calculation was conducted through the function cpa.count in ‘clusterPower’ package in R^37^. The ICC for the current study was identified as 0.01 based on prior research^38^. A detectable effect size of *r* = 0.1 was set for power analysis to capture small changes in outcome measures. The number of schools fulfilling inclusion criteria in the local area was 84, which means each group involved 42 clusters. With a power of 0.85, the mean number of participants calculated for each cluster (school) was 30. A final sample in each group should thus reach 1260. To account for a withdrawal rate of 30%, the overall minimum sample size was 3600 participants (1800 in each comparison group). A post-hoc power analysis was also conducted (See supplementary for further details).

### Randomization and blinding

The 84 schools were randomly assigned (1:1) to either the intervention group or the control group. Random numbers were generated in the adjusting column using the RANDOM function in Microsoft Excel, with each school having a randomly generated number in its corresponding row. The schools were then ordered by the generated random number from largest to smallest. The 42 schools with larger corresponding numbers were assigned to the intervention group, and the remaining 42 schools with smaller corresponding numbers were assigned to the control group. All participants within the same school were assigned to one group altogether. This process is carried out by researchers whom are independent of this program. Although based on the intervention materials participants might have been able to infer that they received suicide prevention training, they were not blinded. The outcome assessors and statisticians who performed the statistical analyses were blinded to the randomization assignment. The allocation process was managed by two research team members PC and DL. All participants within the same school were assigned to one of the groups altogether, no stratification was applied. After group allocation, DQ, XZ, DL, BL, and DC were responsible for enrolling schools and trainer teachers into the intervention.

### Ethics & Inclusion statement

#### Ethical approval

was granted by the Institutional Review Board of Tsinghua University (Project No: 20220128). Participants provided informed consent after receiving a complete description of the study. They were briefed on the study procedures and informed of their right to withdraw at any time. Participants were advised to leave the venue if they felt distressed during the intervention and were referred to professional help if necessary.

### Outcomes

#### Primary Outcomes

The four primary outcomes included the stigma against suicide, literacy of suicide, perceived competence, and willingness to intervene against suicide. Details of the measurement scales are below.

#### Stigma of Suicide Scale

The Chinese version of the 5-item short form of the Stigma of Suicide Scale (SOSS) was used to measure participants’ stigmatized attitudes toward suicidal individuals^39^. Participants indicated their attitudes toward each item using a 5-point Likert scale from 1 (strongly disagree) to 5 (strongly agree). The Chinese short form SOSS has been shown to have good psychometric properties for measuring the stigma against suicide in the Chinese population^40^. Higher total scores indicate more severe stigmatizing attitudes.

#### Literacy of Suicide Scale

The Chinese version of the 11-item Short Form Literacy of Suicide Scale (LOSS) was used to measure participants’ suicide literacy^40^. The LOSS measures one’s literacy regarding the nature, symptoms, and help-seeking methods of suicide by having participants indicate whether they think a statement is true^41^. Participants indicated their responses for each item by choosing from True, False or Not sure, with a correct answer awarded 1 point. The Chinese short form LOSS has shown good psychometric properties for measuring suicide literacy among Chinese people^40^. Higher scores indicate greater literacy of suicide.

#### Perceived Competence, and Willingness to Intervene Against Suicide Questionnaire

The Chinese version of the Willingness to Intervene Against Suicide Questionnaire (WIS) was used to measure participants’ perceived competence and willingness to intervene with students at suicidal risk^42^. The WIS consists of four subscales. In the current study, the 20-item Perceived Behavioral Control Subscale was used to measure participants’ perceived competence to intervene, and the 22-item Willingness to Intervene Subscale was used to measure participants’ willingness to intervene with students at suicidal risk. Participants used a 5-point Likert scale to indicate whether they felt confident or were willing to intervene with a suicidal student through different methods in the perceived behavioral control subscale and the willingness to intervene subscale, respectively. The WIS has shown good psychometric properties for measuring perceived competence and willingness to intervene with a student at suicidal risk^43^. Higher total scores indicate greater perceived competence and willingness to intervene, respectively.

### Secondary Outcome

#### Gatekeeper Behaviors

The study measured participants’ gatekeeper behaviors at 12 months post-intervention. To assess short-term behavioral changes, the intervention group also reported gatekeeper behaviors at the 6-month mark^2,24^. This measurement were conducted using four individual questions that have been adjusted from previous studies to enable a rigorous comparison^44,45^, considering the lagging nature of the observation period for behavior changes. Percentage scores were calculated for each intervention method (e.g., Talk to the students, Talk to the parents, referred to professional help) by comparing the number of students they intervened with to the number of students they identified as at risk for suicide. These scores ranged between 0 and 100%, with a higher percentage indicating a higher frequency of gatekeeper behaviors.

#### Overall Satisfaction:

Overall satisfaction was collected from teachers. Example items include: *Item1: how satisfied are you with the training? Item2: How well did you grasp the content? Item 3: Knowledge Gained: How much did you learn? Item4: Recommend to Others: Would you recommend this training?* Scores for each item ranged from 0 to 5, with higher scores indicating greater satisfaction with the program^46^.

### Statistical Analysis

All statistical analysis was conducted through R 4.1.0. Following the principle of intention to treat (ITT), the missing data for primary outcomes were imputed using multivariate imputation (MI) with the multivariate imputation by chained equations (MICE) package in R^47^. Demographic data and outcome variables were analyzed using baseline univariate between group tests. Two independent sample *t* tests were used to compare the means of outcomes with normal distribution considering the control group and the experimental group. For categorical variables, the Fisher’s exact test was used also considering intervention versus control groups. Descriptive data were presented as mean (*SD*) and *n* (%) as appropriate.

The primary outcomes were conducted through Generalized Estimating Equations (GEE) modeling while controlling for the covariates^48^. The GEE approach allows for the incorporation of the dependence between the observations of the same individual resulting from repeated measures carried out over time. Although this model assumes a normal marginal distribution, the GEE allows the assumption of normality to be relaxed in the distribution of dependent variables^48^. In verifying the time and group effect, we considered four time points (baseline, post-training, and 6- and 12-month follow-ups) and accounted for potential false positives using a false discovery rate (FDR) correction. An autoregressive correlation structure was selected for these repeated measures. Further, to capture the magnitude of change, we calculated the effect sizes of each outcome, using Cohen’s *d,* which represents the between-group difference from baseline to follow-up (6 months and 12 months) and the relative efficacy of LGTP compared to the waitlist control. Based on Cohen^49^,Cohen’s d can be interpreted as small (0.20), medium (0.50), and large (0.80).

In a secondary analysis, two independent sample t-tests were used to compare the means of gatekeeper behaviors outcomes between the control group and the intervention group at the 12-month follow-up. It is worth noting that the data used in here was not imputed to preserve the observation of real behaviors. We also did the post-hoc power analysis to adjust the sample size (see the details in supplementary).

## Data Availability

All inquiries regarding data access should be directed to the corresponding authors. Data sharing is permissible exclusively for research or academic purposes, subject to data use agreements.

## Abbreviations

TTT: Train-the-Trainer
QPR: Question, Persuade, Refer
RCT: Randomized Controlled Trial
LOSS: Literacy of Suicide Scale
SOSS: Stigma of Suicide Scale
